# Evaluation of Novel Compatibility Strategies for Improving the Performance of Recycled Low-Density Polyethylene Based Biocomposites

**DOI:** 10.3390/polym13203486

**Published:** 2021-10-11

**Authors:** Mahmoud M. A. Nassar, Ishaq Sider

**Affiliations:** 1 College of Applied Professions, Palestine Polytechnic University, Wadi Alhareya, Hebron P.O. Box 198, Palestine; 2 Department of Mechanical Engineering, Palestine Polytechnic University, Wadi Alhareya, Hebron P.O. Box 198, Palestine

**Keywords:** biocomposites, chemical crosslinking, compatibilisation, interfacial adhesion, olive pits, recycled polymer

## Abstract

The interfacial compatibility of the natural filler and synthetic polymer is the key performance characteristic of biocomposites. The fillers are chemically modified, or coupling agents and compatibilisers are used to ensure optimal filler-polymer compatibility. Hence, we have investigated the effect of compatibilisation strategies of olive pits (OP) flour content (10, 20, 30, and 40%wt.) filled with recycled low-density polyethylene (rLDPE) on the chemical, physical, mechanical, and thermal behaviour of the developed biocomposites. In this study, we aim to investigate the filler-polymer compatibility in biocomposites by employing novel strategies for the functionalisation of OP filler and/or rLDPE matrix. Specifically, four cases are considered: untreated OP filled rLDPE (Case 1), treated OP filled rLDPE (Case 2), treated OP filled functionalised rLDPE (Case 3), and treated and functionalised OP filled functionalised rLDPE (Case 4). In general, the evaluation of the performance of biocomposites facilitated the application of OP industrial waste as an eco-friendly reinforcing agent for rLDPE-based biocomposites. Furthermore, surface treatment and compatibilisation improved the properties of the developed biocomposites over untreated filler or uncoupled biocomposites. Besides that, the compatibilisers used aided in reducing water uptake and improving thermal behaviour, which contributed to the stability of the manufactured biocomposites.

## 1. Introduction

The demand for the production of environmentally friendly materials for engineering applications has increased over the last decade [1,2]. Green composites are one such type of composites that are an important substitute for existing polymers and conventional composites. Additionally, biocomposites have a long life and are more essential to the environment than conventional composites [3,4]. Materials made of biocomposites provide advantages, such as light weight, comparable strength, thermal stability, chemical and corrosion resistance, and design flexibility [5,6]. An alternative to biocomposites is eco-friendly composites that are developed from natural fillers and recycled synthetic polymers. Since they are manufactured from waste materials, Eco-friendly composites do not harm the environment and are effective in reducing the greenhouse effect and producing greener biocomposites [7,8]. This reduces the amount of wood used in wood plastic composites by recycling the industrial and agricultural waste rather than burning or burying it. [9,10]. To develop advanced and robust technologies and methods, as well as educate the global market on the benefits of re-use and recycling, additional development is essential in the recycling sector [11,12]. In addition to being 40% less expensive than virgin resin, recycled polymer is also cost-effective and protects the environment [13].

Indeed, filler-polymer compatibility, interfacial bonding, and matrix strength are the main parameters that affect the strength of biocomposites [14]. Generally, the performance of polymer composites reinforced with natural fillers degrades due to the weak interfacial adhesion between the filler and polymer. Hence, a natural filler or synthetic polymer that has better surface properties and functionality can help alleviate this problem [15,16]. Moreover, the properties of natural fillers are inconsistent because they depend on the plant species, origin, environment surrounding the plant, and methods of extraction [17]. Thus, it is essential to implement physical, chemical, or biological methods to modify the natural filler surface and reduce the filler inconsistency. Basically, the surface impurities are completely removed and amorphous biomass content, such as lignin is partially removed. Hence, the amount of cellulose and hemicellulose after treatment has a great impact on the mechanical properties of the biocomposites [14,18]. As a result, the addition of treated natural fibres to a synthetic polymer improves its mechanical and tribological properties. However, this performance is affected by the type, fraction or treatment of the filler, type of polymer, or the employed manufacturing process [19,20].

However, to improve the filler-polymer interfacial adhesion, physical adhesion, mechanical interlocking, chemical bonding, or crosslinking are normally employed [21,22,23]. When implemented in biocomposites, compatibilisers act as an intermediary for enhancing the quality of filler-polymer chemical bonding or crosslinking and productivity of biocomposites [24]. Compatibilisers and coupling agents facilitate optimal stress transfer by reducing the filler surface hydrophilisation or by creating chemical crosslinking between the filler and polymer matrix [25]. A wide variety of chemicals, such as silanes, isocyanates, and malleated polyolefin have been used as compatibilisers [26,27,28]. Furthermore, the addition of isocyanate group to the polymer surface improves the interfacial interaction between the filler and polymer, thus improving the mechanical properties [29]. In general, the mechanical properties of the compatibilised biocomposites are better than the industrial properties owing to an improvement in shearing at the filler-polymer interface and uniform stress distribution throughout the structure of biocomposites. Thus, it can be observed that composites are efficient in the production of packaging materials and commodity products [30,31].

Owing to their long lifespan and application in products with short lifespans, plastic polymers are one of the reasons for the current environmental problems [32,33]. Every year, over 500 billion plastic bags mostly made of low-density polyethylene (LDPE) are used around the world. As a result, the amount of plastic waste is likely to increase owing to inadequacies in plastic reuse, recycling, and waste management practices [34,35]. Therefore, this study aims to assess the properties of natural filler-based polymer biocomposites derived from recycled OP and recycled LDPE (rLDPE) using compatibilisation strategies. Additionally, it explores the influence of filler content, chemical treatment, and compatibilisers on the performance of the developed biocomposites. To evaluate the effect of the newly established compatibilisation strategies between OP and rLDPE matrix, we have thoroughly investigated the chemical, physical, mechanical, and thermal properties of the developed biocomposites.

## 2. Materials and Methods

### 2.1. Chemicals and Modifiers

The different types of chemicals and modifiers used in this study are as follows: maleic anhydride (MAH, 95%, Sigma-Aldrich, St. Louis, MO, USA) and 2-Isocyanatoethyl methacrylate (IEM, 98%, Sigma-Aldrich, St. Louis, MO, USA) are used as functional groups; ceric ammonium nitrate (CAN, Sigma-Aldrich, St. Louis, MO, USA); dicumyl peroxide (DCP, 98%, Fisher, Portsmouth, NH, USA) is used as an initiator, acetone (99%, Sigma-Aldrich, St. Louis, MO, USA) is used as a solvent. All the chemicals were used as received without any modification, and all the functionalisation experiments were performed in a heterogenous mixture (polymer and/or filler and solvent) in a round bottom flask with a magnetic stirrer equipped with a condenser.

### 2.2. Filler and Polymer

The rLDPE powder was provided by Suzhou Poks Machinery Co., Ltd. (Suzhou, China) and the properties of rLDPE are shown in Table 1 [36]. As stated previously in a recent article, the usage of polymer powder aids in obtaining a greater efficiency of filler dispersion in the matrix [37]. The OP residues were provided by local industry in Palestine. First, the OP flour was prepared as explained in an earlier study [36]. The raw powder used without any modification is denoted by OP-UT. The treated powder via 5% NaOH is denoted by OP-T. The treatment improved the exposure of hydroxyl group (OH) of the biomass content by removing the surface impurities of the filler, and thus it can be facilitated in chemical functionalisation and crosslinking with the matrix polymer.

### 2.3. Chemical Functionalisation of Filler and Polymer

#### 2.3.1. rLDPE-*g*-IEM

**Scheme 1:**
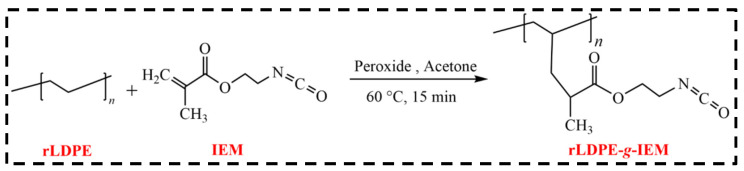
Schematic of grafting reaction of IEM onto rLDPE chains in the presence of DCP.

Polymer powder (rLDPE) is immersed in acetone to prepare a mixture DCP (0.25 wt.% with reference to rLDPE) that is used as an initiator. IEM (0.5 wt.% with reference to rLDPE) used as a functional group was added to the mixture and stirred at 60 °C for 15 min. The product was then filtered and washed with distilled water to remove excess chemicals. The modification reaction procedure is depicted in Scheme 1. It can be observed that the stability of the isocyanate group residing on the rLDPE surface is adversely affected by absorbed moisture that reduces its reactivity and efficiency. Hence, blending of OP-T with rLDPE should be completed immediately after functionalising rLDPE. 

#### 2.3.2. (OP-T)-*g*-MAH

**Scheme 2:**
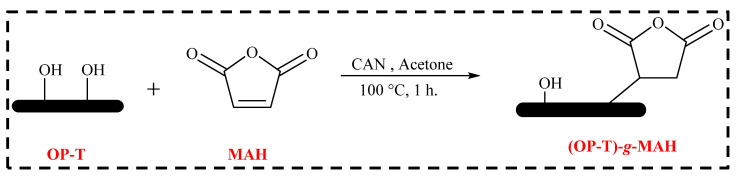
Schematic of grafting reaction of MAH onto OP-T chains in the presence of CAN.

The synthesis was carried out according to the procedure reported in one of the previous studies [38]. The reaction between OP-T and MAH with CAN as an initiator resulted in graft copolymer as shown in Scheme 2. OP-T is mixed with MAH (5-folds with reference to OP-T), CAN (50 wt.% with reference to OP-T), and (200 mL) acetone. The mixture is stirred for an hour, filtered, and washed with distilled water to remove any impurities. Finally, the product is dried at 105 °C for 24 h.

### 2.4. Composite Fabrication

**Figure 1:**
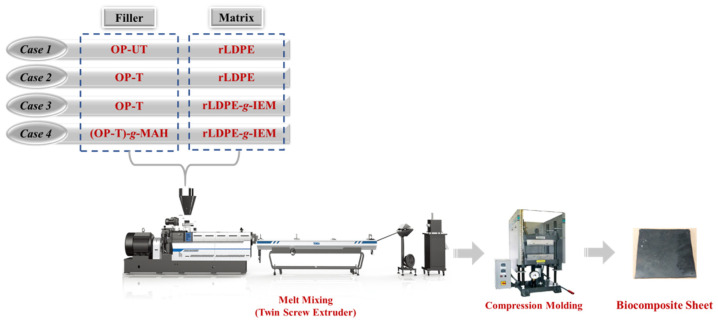
Graphical demonstration of classifications and production of developed biocomposites.

**Scheme 3:**
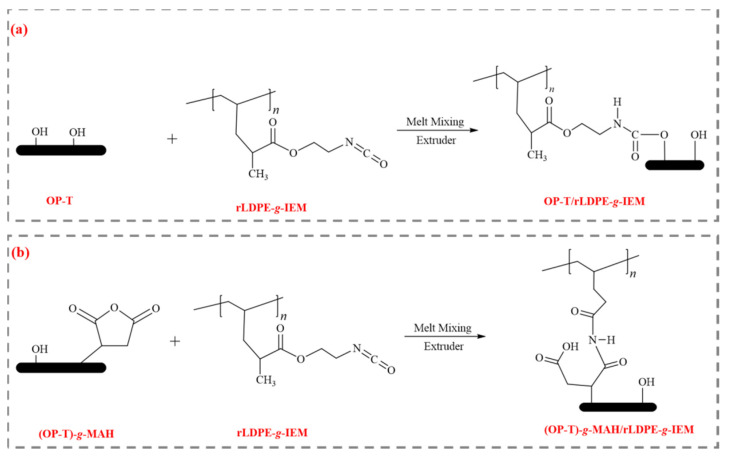
Proposed compatibilisation strategies of (**a**) OP-T/rLDPE-*g*-IEM, and (**b**) (OP-T)-*g*-MAH/rLDPE *g* IEM.

In this study, we have implemented melt mixing followed by compression moulding to fabricate the composite panels. The mixing process and production of biocomposite granules was conducted using a twin-screw extruder (Tengda TSH-35P, Nanjing, China) with 10 consecutive heating zones at a temperature range of 200–220 °C and with screws rotating at a speed at 200 rpm. Then, a stainless-steel mould (230 × 230 × 2 mm) in the compression moulding machine (Carvar, Wabash, IN, USA) was filled with the mixture and compressed between two heated plates at 190 °C under a pressure of 40 MPa. The compression was maintained for 15 min before the heating elements were switched off to let the panel cool down. Then, they were cured by sprinkling tap water on the outer area of the heating plates of the compression moulding for 2 min. Figure 1 shows a graphical representation of the fabrication process and biocomposite sheets. Scheme 3a,b show the compatibilisation strategies proposed for OP-T/rLDPE-*g*-IEM and (OP-T)-*g*-MAH/rLDPE-*g*-IEM. The isocyanate group (NCO) at the rLPDE surface reacts with OH at the treated filler surface as in Case 3 (Figure 1). Similarly, the NCO group reacts with MAH group at the filler surface as in Case 4 (Figure 1). Hence, mechanical interlocking due to filler surface treatment and chemical crosslinking due to functionalisation occurs, thus improving the filler-polymer compatibility.

### 2.5. Characterization

#### 2.5.1. Chemical Characterization

The variation in chemical compositions of the fillers and developed biocomposites were assessed using Fourier transform infrared (FTIR) spectrometer (Agilent, Santa Clara, CA, USA). A total of 64 scans were performed on the samples in a range of 400–4000 cm^−1^ at ambient temperature.

#### 2.5.2. Physical Properties

A densitometer was used to measure the density of the biocomposite samples (Model: MZ-A300, Shenzhen Qun Long Instrument Equipment, Shenzhen, China). At a temperature range of (23 ± 2 °C), a 2 g specimen sample was immersed in distilled water and the volumetric change in water was measured. The average density of five samples was measured and reported.

Procedures from the ASTM D570 standard were used to evaluate the water absorption of the produced biocomposites. Five dried samples were immersed in distilled water for 24 h at room temperature. The proportion of water content absorbed by the biocomposite specimens was then confirmed by observing the sample weights before and after soaking in water.

To assess the crystallinity of the natural fillers and biocomposite sheets, the samples were scanned using an X-ray diffractometer (Rigaku Corporation, Tokyo, Japan) at room temperature. The measurements were made at 40 kV and 20 mA using a detector placed on a goniometer scanning scale from 10–60° at a scanning speed of 5° min^−1^ using monochromatic CuK radiation (λ = 1.5406 nm). The crystallinity degree was then determined as described in previous work [39,40].

Furthermore, the melt flow index (MFI) values of the produced biocomposites were compared with the neat polymers (DRK208B Plastic Melt Flow Index tester, Qingdao, China) at 190 °C with a standard weight of 2.16 kg following the ASTM D1238 standard procedure [41]. The average MFI for three samples was then calculated.

#### 2.5.3. Mechanical Properties

The ASTM D638 standard was used to determine the tensile characteristics of the developed biocomposites. Tension tests were performed using a universal testing machine (Tinius Olsen 10 kN, Redhill, UK) with a crosshead speed of 5 mm/min. Specimens were held vertically between the grips of the testing machine, which were uniformly and securely adjusted to avoid slippage, and the gauge length was maintained at 30 mm. The experiments were carried out at room temperature. Five specimens of each kind of developed biocomposites were tested and the average tensile characteristics were reported.

#### 2.5.4. Thermal Properties

The thermogravimetric analysis (TGA) and derivative thermogravimetric (DTG) plots were analysed using a thermogravimetric analyser to evaluate the thermal behaviour (TGA Q 500 TA Instrument, New Castle, DE, USA). The samples were heated in an inert environment, deposited in an aluminium pan, and heated in the range of 20–600 °C. This was followed by an analysis of the plots with TA Universal Analysis software (v5.5.24).

## 3. Results and Discussion

### 3.1. Chemical Characteristics

**Figure 2:**
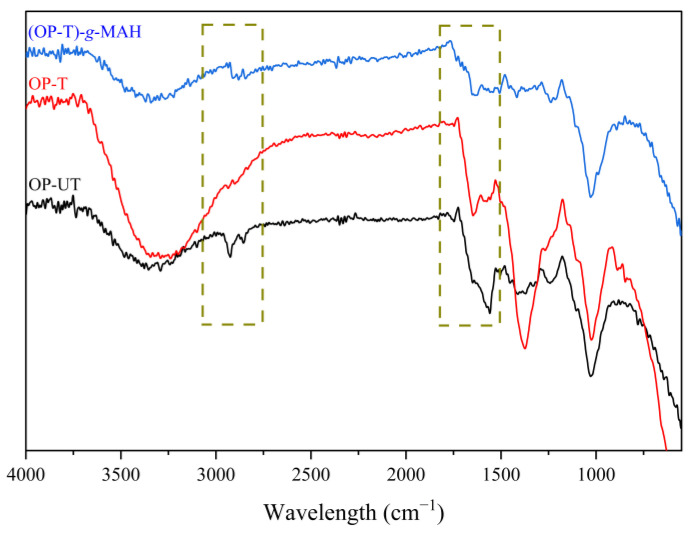
FTIR spectra of the untreated filler (OP-UT), treated filler (OP-T) and functionalised filler ((OP-T)-*g*-MAH).

Figure 2 shows the characterisation of the chemical variations of filler treatment and MAH grafting confirmation on the OP using FTIR. Compared to OP-UT, the structural integrity of OP-T and (OP-T)-*g*-MAH fillers is retained as confirmed by the chemical composition. The absorption bands of the filler in all the forms are almost the same in any of the FTIR observations. According to FTIR, carboxyl/aldehyde groups in acids found in hemicellulose, carbonyl/carboxyl groups in pectin, lignin segments, and aliphatic fatty acids found in fibre wax are all represented by a 1746 cm^−1^ band [42]. After treatment with NaOH, the primary changes are identified by the removal of the peak at approximately 1746 cm^−1^ indicating that the surface impurities of the filler were eliminated. Thus, the topography and functionality of the surface are enhanced [16,43] as indicated by the increased intensity of the OH peak at 3300 cm^−1^, which promote access to the OH groups [44]. Furthermore, OP-UT shows a series of bands in the 2950–2800 cm^−1^ range attributed to C–H asymmetric and symmetric stretching vibrations in the cellulose and hemicellulose groups [42]. The peak at 2861 cm^−1^ decreased substantially, suggesting that the residual OP-T composition was largely made up of cellulose [45,46]. Since the surface impurities were effectively removed by the chemical treatment, the OH of cellulose is exposed. Because of the resulting roughening of the filler surface and increased exposure of the OH group to the cellulose surface, the filler functionality and interfacial adhesion to the compatibiliser at the polymer surface are enhanced. The functionalisation of (OP-T)-*g*-MAH indicates absorption at 1705 and 1735 cm^−1^ owing to the asymmetric stretching vibration of the newly introduced C=O groups, which demonstrates a successful grafting of MAH on the surface of OP-T. This agrees with the findings of Lisperguer et al. [45].

**Figure 3:**
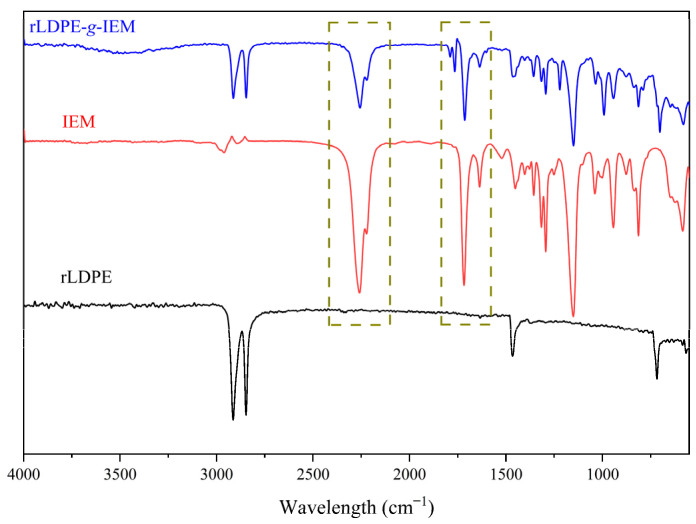
FTIR spectra of rLDPE, IEM, and rLDPE-*g*-IEM.

The FTIR of pure IEM (Figure 3) shows absorption bands at 1718 and 2260 cm^−1^, which are characteristic of C=C stretching vibration (vinyl saturation) and NCO group, respectively. After functionalisation, the FTIR spectra of rLDPE-*g*-IEM display stretching absorption at 2262 cm^−1^ due to N=C=O and peaks at approximately 1760 and 1798 cm^−1^ are associated with the grafted DCP [46]. This confirmed that the functionalisation was successfully accomplished and the N=C=O group was grafted onto the rLDPE surface as presented in Figure 3. The grafted N=C=O moiety is highly reactive towards the OH group on the OP-T surface. Therefore, the subsequent reaction between N=C=O moiety on rLDPE and OH group on OP-T creates a urethane bond (carbamate esters) between the filler and polymer matrix [26,29].

**Figure 4:**
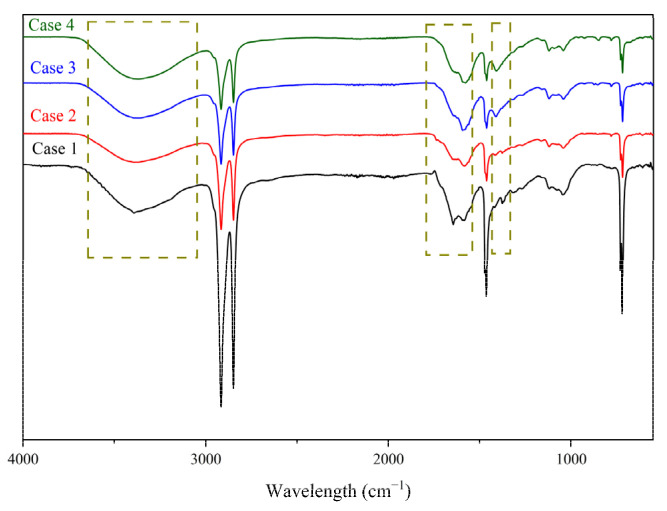
FTIR spectra of the developed biocomposites.

The FTIR of the chemical compositions of the four biocomposites generated after melting the fillers and polymer matrix are presented in Figure 4. Biocomposites produced with OP-UT and OP-T exhibit no substantial changes in the chemical composition; however, the peak intensity is clear and intense when the OP-T is employed as a filler [36]. In contrast, minor changes are observed when compatibilisers (Case 3 and 4) are used. It must be noted that the characteristic peak at 2262 cm^−1^ due to N=C=O group disappeared in the product [47] with a corresponding increase in the carbonyl peak (C=O) in the region with absorption bands at 1600–1720 cm^−1^. This large band (typically, between 1620 and 1760 cm^−1^) is very complicated and difficult to interpret because it encompasses several individual and overlapped absorption bands [48,49]. In addition, the new peak that appears at 1410 cm^−1^ is associated with the C–N stretching band. These findings suggest the formation of urethane [–NH-C(O)=O] [50]. However, the successful crosslinking between (OP-T)-*g*-MAH and rLDPE-*g*-IEM, which indicates the production (OP-T)-*g*-MAH/rLDPE-*g*-IEM based biocomposite, was confirmed by using FTIR, and the comparative spectra is presented in Figure 4 (Case 4). After melt mixing of (OP-T)-*g*-MAH and rLDPE-*g*-IEM, a characteristic peak at approximately 1500–1650 cm^−1^ is observed in the product, which might be attributed to the C=O and N–H functionalities that are related to amide I and II formations, respectively [51]. Hence, the amide linkage formation is confirmed owing to the crosslinking reaction between MAH and NCO on the filler and polymer surfaces, respectively [50]. Thus, the successful coupling mechanism between the functionalised polymer and filler is confirmed as proposed in Scheme 3a,b.

### 3.2. Tensile Properties

**Figure 5:**
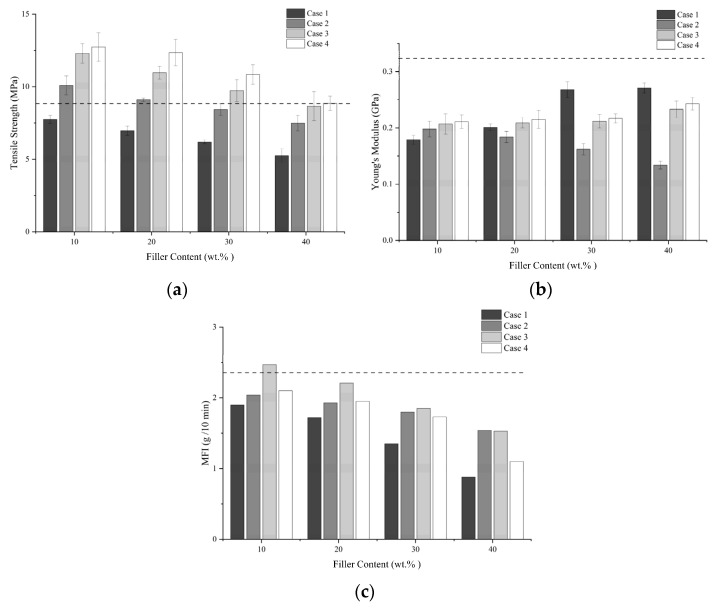
Tensile properties of the developed biocomposites: (**a**) tensile strength, (**b**) Young’s modulus, and (**c**) MFI values of the developed biocomposites (The dashed line represents the characteristics of LDPE).

Tensile properties and MFI of the developed biocomposites for the four cases are presented in Figure 5. In general, biocomposite tensile properties depend on the filler content, filler treatment, and the use of compatibilisation. With an increase in the filler content, the mechanical performance degrades corresponding to an increase in discontinuous regions related to the particulate filler; hence, the likelihood of micro agglomeration increases. This indicates that a better fibre-matrix interface is attained following the treatment because of the mechanical interlocking of the polymer-filler at the interface. The overall characteristics of the composites are determined by the adhesion between the matrix and fibre. To improve the performance, a combination of filler treatment and compatibilisers is proposed as demonstrated in the performance of biocomposites in Cases 3 and 4. Using both mechanical interlocking due to filler surface roughness and chemical crosslinking due to compatibilisers, the efficacy of mechanical stress transmission at the filler-polymer interface is demonstrated. The best performance of the developed biocomposites is observed in Case 4 when the filler and polymer are both chemically functionalised. This might be attributed to the improved functionality of the filler owing to the grafted MAH and type of bonding established with the NCO at the functionalised polymer surface.

The Young’s modulus of the developed biocomposites is shown in Figure 5b. Generally, the addition of particulate (rigid) filler induces stiffness in the biocomposites especially for OP-UT filler rLDPE. On comparing the cases of raw and treated fillers, the developed biocomposite using OP-T and compatibiliser recorded a lower value of Young’s modulus. Additionally, the modulus increases with an increase in the filler content as presented in Figure 5b. This is because, Young’s modulus is a nonlocal material property; i.e., it is less sensitive to local stress distribution. Thus, adding rigid fillers induces stiffness in the biocomposites. This stiffness is higher in treated filler owing to the treatment due to the removal of non-cellulosic content, which tends to make the filler texture more brittle. This is in agreement with the findings presented by Ismail et al. [52]. The effect of compatibilisers as reported in Cases 3 and 4 show that the value of Young’s modulus improves due to better filler-polymer compatibility and loading transfer at the interphases. Thus, the addition of compatibilisers improved the interfacial strength, and the capability of load transfer and resistance to deformation as indicated by an increase in the values of the tensile modulus. Similarly, the use of treated OP fillers and compatibilisers in the rLDPE matrix develops higher MFI properties compared to OP-UT filler rLDPE (Case 1) as presented in Figure 5c. This indicates that rLDPE with the OP filler exhibits better compatibility with an improvement in MFI. The highest performance of the developed biocomposites is recorded in Case 3 followed by Case 4, which may be an attribute of the bonding nature and grafted functional group on the surface. Hence, additional research on the rheological properties of functionalised materials is essential to get a deeper insight into the functionalisation effect of the biocomposite ingredients, which was not available in our lab during the research. 

### 3.3. Physical Properties

#### 3.3.1. Density and Water Absorption

**Figure 6:**
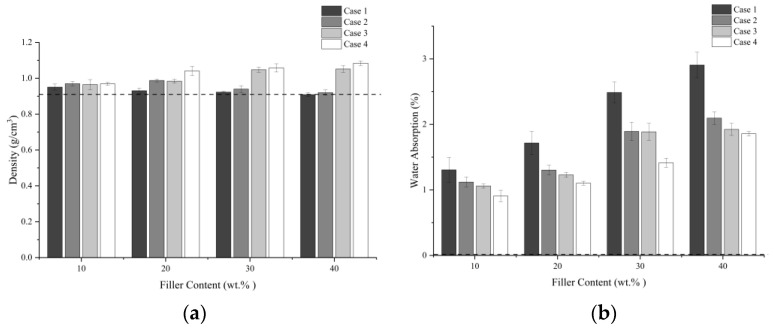
(**a**) Density and (**b**) water absorption of developed biocomposites (The dashed line represents the characteristics of LDPE).

Density and water absorption of the biocomposites have an impact not only on the filler content but also on the nature of the filler-polymer interface. Better adhesion among the biocomposite ingredients indicates fewer subsurface voids and porosity, which ensures that the material is less dense and subjected to fewer subsurface cracks. This improves the biocomposite performance in terms of water repellent behaviour [53]. Figure 6 effectively demonstrates this behaviour of the developed biocomposites. Additionally, the developed biocomposites using compatibilisers (Cases 3 and 4) exhibit increased density as the water absorption decreases, which indicates an improvement in filler-polymer adhesion and compatibility. Moreover, the results indicate a remarkable improvement in the physical properties of the developed biocomposites for OP-T and compatibilisers. However, when the compatibilisers are not used, OP-UT based biocomposites (Case 1) showed lower density followed by the OP-T based biocomposites (Case 2), and higher water absorption was recorded for OP-UT based biocomposites (Case 1) followed by the OP-T based biocomposites (Case 2). This confirms the importance of treatment to improve the performance of biocomposites. The treatment enhances the filler hydrophobicity as a result of the surface impurities and amorphous biomass content removal after the treatment. 

#### 3.3.2. X-ray Diffraction

**Figure 7:**
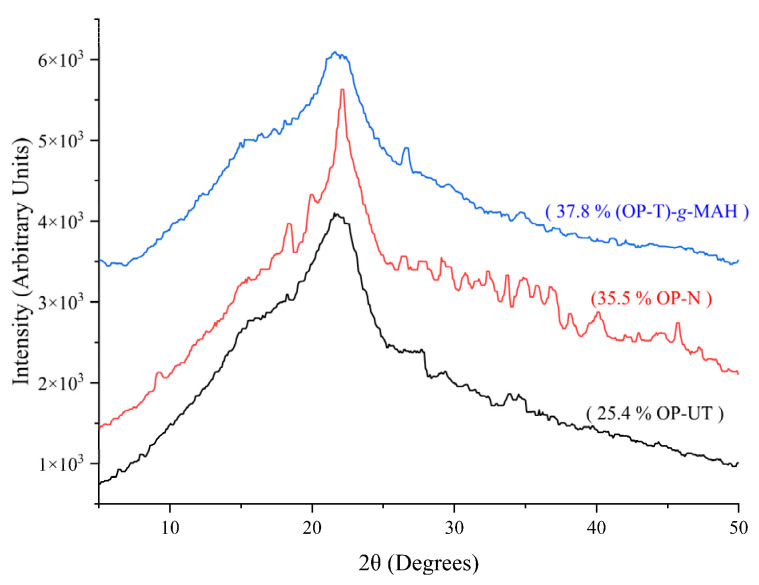
XRD patterns of OP-UT, OP-T, and (OP-T)-*g*-MAH.

The crystallinity of the natural filler after treatment and functionalisation is measured using X-ray diffraction (XRD) as presented in Figure 7. According to the previous literature [39,40], I_002_ in the range of 21.5–22° is the maximum intensity of the crystalline region, which corresponds to cellulose I. And, I_am_ in the 2θ angle range of 18–19° is the maximum intensity of the non-crystalline (amorphous) region, which corresponds to cellulose II. The XRD patterns indicated in Figure 7 show that the crystallinity degree of the OP-T increases by 39.8% compared to the OP-T. This suggests that the applied treatment can partially remove the amorphous biomass without affecting the crystalline biomass, which agrees with the FTIR observations. Meanwhile, the functionalisation of OP-T increases the crystallinity by 56.7 and 12.1% compared to the untreated and treated OP, respectively. This is due to the grafted MAH or the removal of amorphous biomass due to the solvent or chemical used during the functionalisation process. 

**Figure 8:**
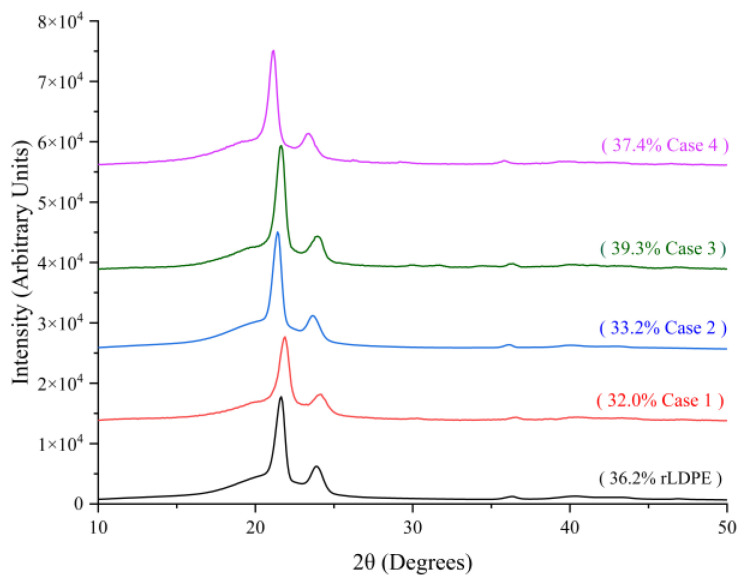
XRD curves of developed biocomposites at 20% filler content compared to neat rLDPE.

Figure 8 presents the typical XRD patterns at 20% filler for the cases in comparison to the neat polymer, and Table 2 summarises the degree of crystallinity of the developed biocomposites at different filler content. The XRD pattern of rLDPE (Figure 8) shows two peaks at a 2θ value of 21.6° and 23.8° that correspond to the crystalline and amorphous region of the polymer. This is in agreement with the pattern of the virgin LDPE according to Carotenuto et al. [54]. According to the research, the recycling process does not affect the main characterises of the polymer. Moreover, the biocomposite patterns showed a similar pattern of the polymer indicating that the filler was embedded in the matrix and did not affect the main crystalline characteristics of the polymer even for a high filler content (40 wt.%). However, the effect of filler content and compatibilisation on crystallinity is clearly indicated in Table 2. It is observed that the filler content is inversely proportional to the degree of crystallinity in all the cases due to the structure discontinuity because the filler is in particulate form. When a load is applied, crystallinity also indicates how well molecular chains are structured and slide over one another. As a result of the increased crystallinity, the load transfer capacity among composite ingredients improved, indicating better filler/polymer compatibility [42]. However, for treated filler, there is a slight increase in the degree of crystallinity, which indicates improved interfacial bonding of the filler-polymer surfaces. The improvement was detected in Cases 3 and 4 as well, where the compatibilisers are used, especially in Case 4. This indicates an improvement in the filler-polymer intermolecular adhesion because of chemical crosslinking and bonding. Moreover, the filler crystallinity affects the biocomposite crystallinity as seen in Case 4 where the filler crystallinity was higher after the treatment and functionalisation. Furthermore, when treated filler and chemical crosslinking are employed with identical filler content, the crystallinity improved. This may be explained as a result of the compatibilizer and chemical crosslinking, which increases the compatibility of the filler-polymer interface.

### 3.4. Thermal Properties

**Figure 9:**
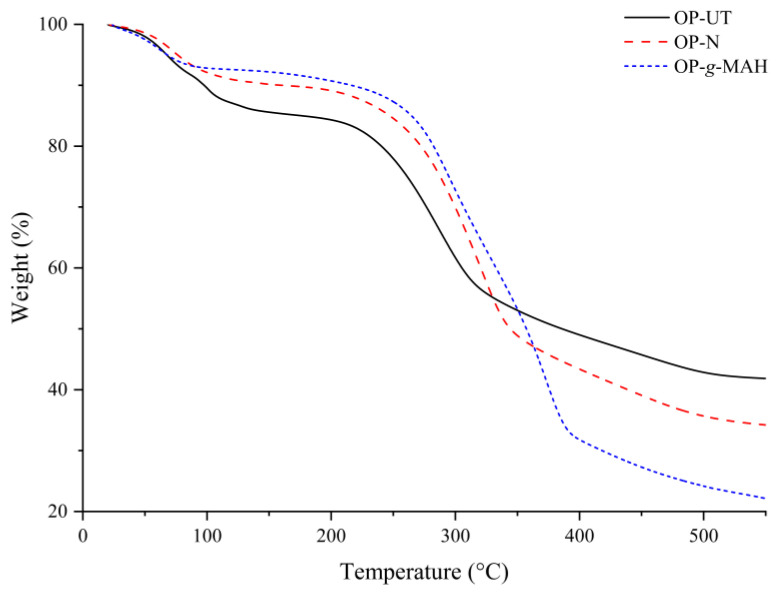
TGA curves of OP-UT, OP-T and (OP-T)-*g*-MAH.

**Figure 10:**
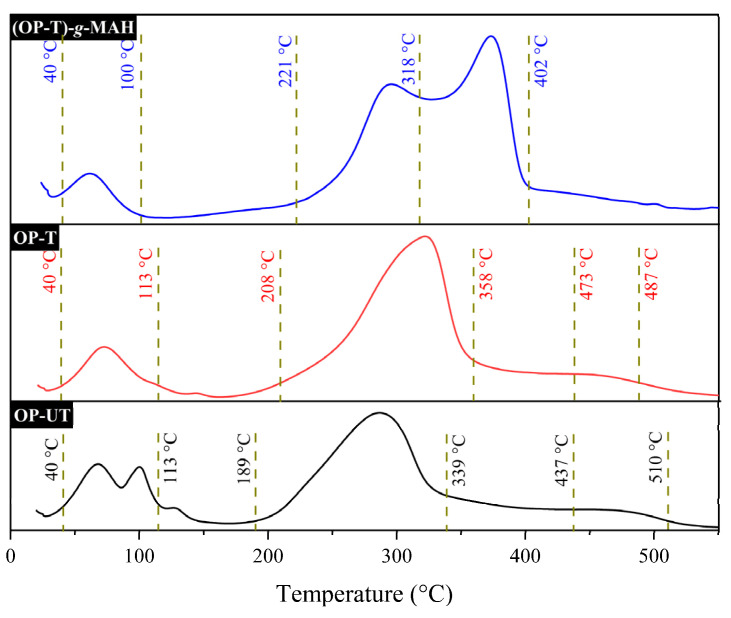
DTG curves of OP-UT, OP-T, and (OP-T)-*g*-MAH.

The thermal behaviour of materials is an essential feature to measure the thermal stability and degradation mechanism of materials at different temperatures that determine the applicability of those materials in various sectors. Hence, TGA/DTG analysis was conducted on the natural fillers and developed biocomposites as shown in Figure 9 and Figure 10. Based on TGA, three mass loss steps are observed in the range of room temperature (23 ± 2 °C) to 550 °C. Natural filler stability sustained up to 190 °C for natural fillers with a slight delay in the decomposition for treated and functionalised fillers. Results of weight loss reveal an evident change in the thermal degradation process of treated and functionalised fillers due to the removal of impurities, partial removal of amorphous content (hemicellulose), and addition of the functional group (MAH). The incorporation of the MAH group positively contributes to the thermal stability of the filler by introducing new molecules that foster intermolecular interactions, which agrees with the findings of a previous study [16].

When the temperature is above 450 °C, the fillers are thoroughly degraded so that the residual mass is constant with increasing temperature. The remnant ash yield (residue) at the end of the thermal analysis for the sample is approximately 41.9, 34.3, and 22.2% for OP-UT, OP-T, and (OP-T)-*g*-MAH, respectively. Higher residue is associated with rich content of the amorphous biomass especially lignin, which is observed in OP-UT [55], whereas the residue in OP-T and (OP-T)-*g*-MAH is decreased, which indicates that the filler is rich in cellulose due to the treatment and functionalisation. This leads to the removal of non-cellulosic materials (hemicellulose and lignin) from the raw fillers, and thus the filler stability is enhanced [56].

For detailed analysis and understanding of the degradation steps of the filler, the DTG analysis is conducted as depicted in Figure 10. For the three types of fillers, the first decomposition (under 140 °C) zone is associated with dehydration. When the temperature increases, each filler exhibits a distinguished decomposition zone, which highlights the treatment and functionalisation effect of the thermal degradation process. While the major mass loss of the fillers begins at approximately 200 °C, the decomposition ends with a delay in the case of treated and functionalised filler. This delay is a clear indication of the elimination of potential wax and surface impurities along with the partial removal of amorphous content. Indeed, the main decomposition zone is associated with the hollocellulose content (Hemicellulose and cellulose) at approximately 200–350 °C for OP-UT and OP-T. However, distinct peaks of hemicellulose and cellulose degradation are noticed in the case of (OP-T)-*g*-MAH due to the deep incorporation of the MAH group on the cellulose backbone [16]. The final decomposition zone is identified in OP-T and OP-UT only, which denote the degradation of lignin and its remnants after the treatment [57,58]. This peak was not observed in (OP-T)-*g*-MAH, which indicates the least percentage of lignin. This agrees with the XRD findings.

**Figure 11:**
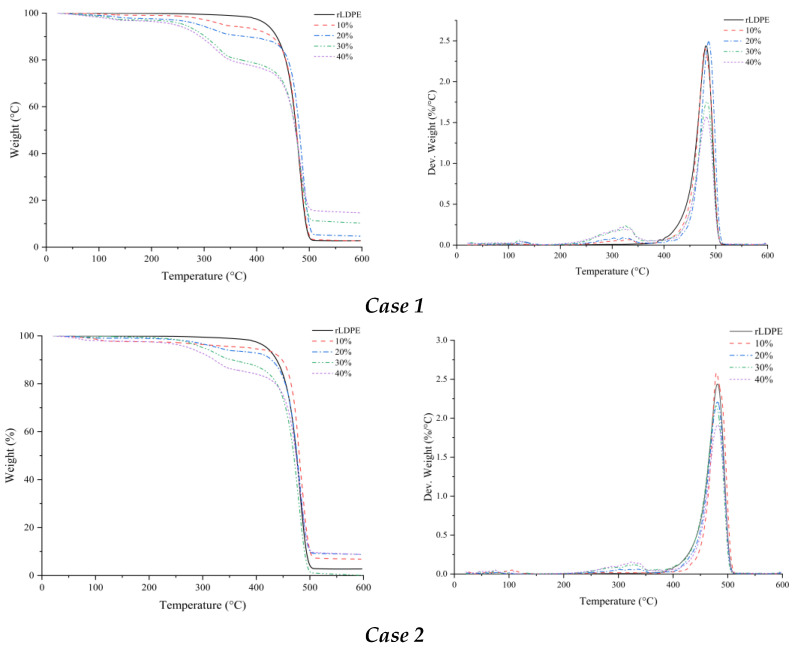

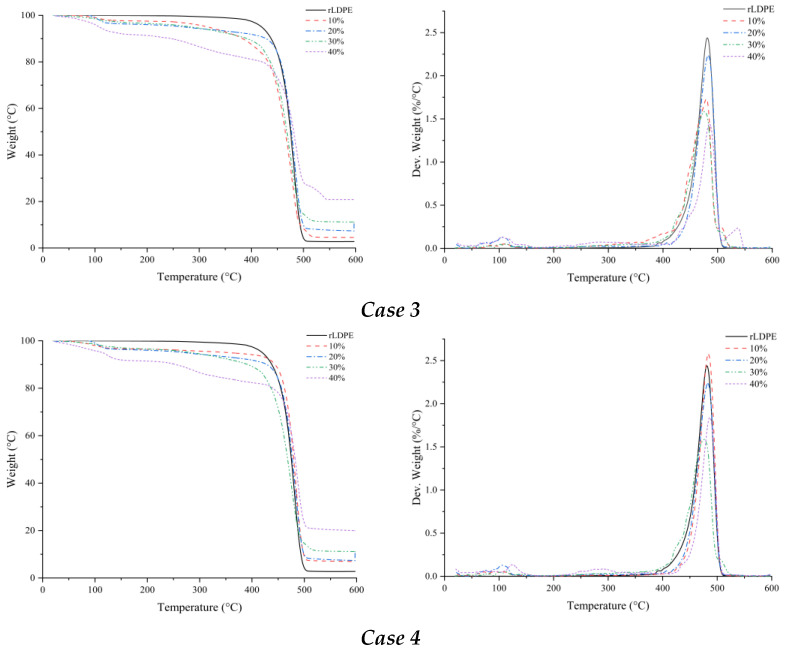
Comparison of TGA/DTG curves of the developed biocomposites with rLDPE.

Figure 11 depicts the comparison of thermal behaviour of the developed biocomposites with rLDPE using TGA/DTG. Three decomposition zones are observed for the biocomposites while only one degradation zone is observed for the polymer matrix. rLDPE contains only one peak; this indicates that there is only one degradation zone from 375–500 °C. In contrast, the developed biocomposites showed two more zones due to the degradation of the embedded filler in the polymer matrix which has been illustrated earlier (Figure 11). In the first zone, weight loss occurs at approximately 110 °C, which corresponds to the internal moisture associated with the filler nature. However, as compared to Cases 1 and 2, Cases 3 and 4 lose more weight at 100 °C, which might be owing to the higher energy required to break down some components of the additional functional groups and chemical bonding that occurs after mixing. Clearly, the second zone is observed between 240–360 °C, which is related to the decomposition of the amorphous biomass especially hemicellulose. This peak is hardly noticed in the case of compatibilisers (Cases 3 and 4) owing to the improved filler-polymer interface bonding, which requires a higher amount of thermal energy to break the bonds. This improves the thermal stability and storage modulus of the biocomposites [21]. Natural fillers, as reported by Lee et al. [21], provide a nucleating site for polymer cold crystallisation, pushing the crystallisation temperature higher as bonding at the interface prevents sliding of polymer chains, delaying the transition from glassy state to rubbery state, and thus improving the material thermal stability, which accords with the findings of this study in Cases 3 and 4. Finally, the major mass loss for the biocomposites is recorded at a temperature range of 400–510 °C, which is attributed to both the rLDPE and biomass content degradation especially cellulose. In general, the slight improvement of thermal behaviour is identified in Case 4 followed by Cases 3 and 2, thus confirming that the use of treated fillers and chemical bonding is beneficial in obtaining thermally stable biocomposites and this behaviour is applicable for various industrial applications [59,60].

## 4. Conclusions

This study proposes two novel methods for improving filler-polymer compatibility and analyzes the chemical, physical, mechanical, and thermal properties. The parameters that affect the performance of biocomposites are filler content, treatment, and filler-polymer degree of compatibility. We used compatibilisers and treated filler to obtain improved performance of the biocomposites. This provided better filler compatibility with polymer matrix, and the formation of micro-voids and sub-cracks was reduced as confirmed by density and water absorption values. The use of compatibilisers was influential in improving the mechanical properties of the biocomposites as indicated by the tensile properties. Additionally, XRD of the fillers along with the bonding nature affected the crystallinity of the developed biocomposites. The presented results indicated that the developed biocomposites are thermally stable and can be viable for various industrial applications.

This research revealed that combining recycled low-density polyethylene with lignocellulosic wastes can result in high-value material. Despite the fact that a greater filler content results in poor overall qualities, the material is suitable in a range of applications where structural specifications aren’t the most important need, and it also meets the desire for eco-friendly materials. In the future, we aim to develop composites with components, such as processed fibre and resin along with fillers. The research efforts will be refined to ensure better use of upgraded materials and procedures for converting them into products. Incorporating tiny amounts of virgin or waste polymers with waste from the same or different polymers and appropriate compatibilisers might result in superior characteristics to the agro-residues.

## Figures and Tables

**Table 1:** Properties of rLDPE.

Property and Unit	Value
Tensile Strength (MPa)	8.78 ± 0.19
Young’s Modulus (GPa)	0.33 ± 0.02
Water Absorption (%)	0.04 ± 0.008
Density (g/cm^3^)	0.902 ± 0.012
MFI (g/10 min)	2.36 ± 0.25

**Table 2:** Summary of degree of crystallinity of the developed biocomposites compared to neat rLDPE.

Filler Content (wt.%)	Case 1	Case 2	Case 3	Case 4
rLDPE	36.2			
10	32.5	37.2	39.7	42.9
20	32.0	33.2	39.3	37.4
30	27.3	30.9	33.2	34.9
40	25.8	30.5	30.3	35.1
